# Everybody's Page

**Published:** 1892-09-24

**Authors:** 


### EVERYBODY'S PAGE.

What strange combinations of employments we hear of sometimes. Lately we heard of a Chinaman who was both doctor and farmer. He was a Christian, and three times daily, before meals, he held family prayers.

An absurd mistake happened in France not long ago. An engine-driver arrived at a station minus his train, which he did not discover he had left behind at a previous station ten minutes before. The station master had given the signal to start, being unaware that the train was not coupled.

Many of the fair sex will welcome the repeal of the rule excluding them from membership of the British Medical Association. Mrs. Garrett Anderson, herself a member before the law of exclusion came into force, seconded the resolution to abolish it, and was most heartily supported.

It is difficult to believe the statements as to modern Chinese cannibalism made by a writer in a Shanghai daily paper. The canabalism is the result of a superstition, and is resorted to for curative purposes only. It is usually induced through filial zeal, the flesh of a child having great supposed healing properties in the case of illness of a parent. The writer of the article dealing with the subject states that a literary graduate in his service cut off a joint of one of his fingers, which he made into a broth mixed with medicine and gave to his mother. It is part of the spell that the recipient of the offering should remain in ignorance of the sacrifice.

At the late meeting of the British Medical Association, Dr. Mason suggested that the difficulty of disposing of town refuse might be overcome by the erection of furnaces to destroy all that was both unwholesome and useless.

A little wholesome terror is very effective. The Ameer of Bokhara finding that sanitary regulations ordered for that city were not being carried out, promptly commanded the execution of every subject not obeying the regulations within one week.

It is somewhere stated that genius rushes like a whirlwind—talent marches like a cavalcade of heavy men and heavy horses—cleverness skims like a swallow in the summer evening, with a sharp, shrill note and a sudden turning. The man of genius dwells with men and with nature; the man of talent in his study, but the clever man dances here, there, and everywhere, like a butterfly in a hurricane, striking everything and enjoying nothing, but too light to be dashed to pieces.

A Swiss doctor claims to have found by experience a novel mode of relief in some affections of the throat and in case of earache. By making the patient yawn two or three times a day, the pain, he states, becomes distinctly lessened. In catarrh of the eustachian tube, the yawning by distending the muscles is said to act as a massage, and by this treatment the affection is frequently cut short. The possibility of so simple a cure should be known to all sufferers from earache. In any case, if no relief is obtained no harm is done, nor would any delay in adopting any other treatment be caused.

